# Cesarean section in Uruguay from 2008 to 2018: country analysis based on the Robson classification. An observational study

**DOI:** 10.1186/s12884-022-04792-y

**Published:** 2022-06-07

**Authors:** Mercedes Colomar, Valentina Colistro, Claudio Sosa, Luis Andres de Francisco, Ana Pilar Betrán, Suzanne Serruya, Bremen De Mucio

**Affiliations:** 1 Montevideo Clinical and Epidemiological Research Unit, Montevideo, Uruguay; 2 Latin American Center for Perinatology, Women and Reproductive Health (CLAP/WR), PAHO/WHO, Montevideo, Uruguay; 3 Department of Quantitative Methods, School of Medicine, Universidad de la República (UdelaR), Montevideo, Uruguay; 4 Department of Obstetrics and Gynecology, Pereira Rossell Hospital, School of Medicine, Universidad de la República (UdelaR), Montevideo, Uruguay; 5 Family, Health Promotion and Life Course, Pan American Health Organization, World Health Organization, Washington, United States; 6 UNDP/UNFPA/UNICEF/WHO/World Bank Special Programme of Research, Development and Research Training in Human Reproduction (HRP), Department of Sexual and Reproductive Health and Research, World Health Organization, Geneva, Switzerland

**Keywords:** Caesarean section, Robson classification, Uruguay

## Abstract

**Background:**

The use of caesarean section has steadily increased, with Latin America being the region with the highest rates. Multiple factors account for that increase and the Robson classification is appropriate to compare determinants at the clinical level for caesarean section rates over time. The purpose of this study is to describe the evolution of caesarean section rates by Robson groups in Uruguay from 2008 to 2018 using a country level database.

**Methods:**

We included the records of all women giving birth in Uruguay (pregnancies ≥22 weeks and weights ≥500 g) with valid data in the mode of childbirth recorded in the Perinatal Information System database between 2008 and 2018. Caesarean section rates were calculated by Robson groups for each of the years included, disaggregated by care sector (public/private) and by geographical area (Capital City/Non-Capital), with time trends and their significance analyzed using linear regression models.

**Results:**

Of the total 485,263 births included in this research, the overall caesarean section rate was 43,1%. In 2018, among the groups at lower risk of caesarean section (1 to 4), the highest rates were seen in women in group 2B (98,8%), followed by those in group 4B (97,9%). A significant increase in the number of caesarean sections was seen in groups 2B (97,9 to 98,8%), 3 (8,36 to 11,1%) and 4 (A (22,7 to 26,9%) and B (95,4 to 97,9%) Significant growth was also observed in groups 5 (74,3 to 78,1%), 8 (90,6 to 95,5%), and 10 (39,1 to 46,7%). The private sector had higher rates of caesarean section for all groups throughout the period, except for women in group 9. The private sector in Montevideo presented the highest rates in the groups with the lowest risk of caesarean section (1, 2A, 3 and 4A), followed by the private sector outside of the capital.

**Conclusion:**

Uruguay is no exception to the increasing caesarean section trend, even in groups of women who have lower risk of requiring caesarean section. The implementation of interventions aimed at reducing caesarean section in the groups with lower obstetric risk in Uruguay is warranted.

**Supplementary Information:**

The online version contains supplementary material available at 10.1186/s12884-022-04792-y.

## Background

Caesarean Section (CS), or C-section is a life-saving procedure when performed timely, appropriately and following precise medical indications. It is also the most common major surgical intervention in many countries [1]. Its prevalence has steadily increased across the globe, particularly in middle and high-income countries, with Latin America and the Caribbean (LAC) being the regions with the highest rates (40,5%) [2]. There are significant inequities in low and middle-income countries, as CS rates are five times as frequent among the wealthiest quintile (median 19,1%, 10,6–33,8 interquartile range (IQR)) of the population versus the poorest quintile (4,1%, 1,9–12,0) [3, 4]. However, a significant proportion of healthy women undergo CS unnecessarily despite the increased risk of serious maternal outcomes with the procedure, and counter to the recommendation to perform it only when the benefits anticipated are clear and offset the increased cost and additional risk associated with the operation [5]. Multiple factors account for that increase [6–9].

According to a systematic review, health professionals’ beliefs are the main determinant in the use of a CS (perception that the procedure is devoid of risk, lack of cooperation and trust among professionals, ideas about women’s preferences). Factors related to the health system also play a role (fear of litigation, medical remuneration structures, policies and existence of clinical practice guidelines), as well as the profile of professionals (convenience, age, gender, status and skills of the professional in charge) [7]. Women’s preferences are also reported as decisive, with studies citing issues such as autonomy and lack of perception of risk [8, 10]. The idea that an in-depth understanding of these determinants at the clinical level requiring the use of a classification system for CS led the World Health Organization (WHO) to conduct a systematic review of the systems used to classify CS in 2011 [11, 12]. It concluded that the Robson classification proposed in 2001 was the most appropriate to systematically evaluate and compare CS rates over time, as well as to account for local and international needs [11, 13–15]. Monitoring the frequency of CS by Robson groups allows a proper evaluation of clinical practice, by considering the obstetric characteristics of women (parity, previous CS, gestational age, onset of labour, fetal presentation, and number of fetuses) [13, 16].

In 2008 Uruguay established a national health insurance system funded by private and public sources. Public sources include mandatory contributions from workers and taxes gathered into the National Health Fund (FONASA). The health providers are: the Collective Medical Assistance Institutions (IAMC), a collection of non-profit private institutions, operating a prepayment system and providing comprehensive health care at all levels to 55% of the registered worker population and their families, including full coverage of obstetric care [17]; public providers include a network of public hospitals (ASSE) and university hospitals, that provide comprehensive health care mainly to low income populations, as well as to military and police forces (42%). Private insurance offers a comprehensive bundle of benefits in exchange for a premium fee. These schemes are aimed at high-income sectors (3%). All healthcare providers (IAMC, public providers, and private insurances) receive per capita payments from FONASA according to the risk of the population covered and care goals established by the Ministry of Health. Private contributions make up out-of-pocket expenses in the IAMC and the payment of the private insurance fee. All health care providers have the equal access to skilled staff, equipment and resources to provide CS.

In Uruguay there has been a progressive increase in the national CS rate, with figures going from 35.5% (2009) to 46.3% (2014), although percentages differ between regions and institutions [18, 19]. In view of this increase, in 2017 the Uruguayan Ministry of Health developed the main guidelines for a strategy aimed at reducing preventable CS, based on Robson categories 1, 2, 3 and 4, which were considered the lowest risk groups [20, 21].

Uruguay monitors obstetric care through the Perinatal Information System (SIP, for its acronym in Spanish) [22]. This system issues automatic reports for the monitoring of obstetric and neonatal events. In 2017, this system registered 98,7% of births in the country [23], making it possible to automatically categorize women based on Robson’s classification system.

The purpose of this study was to describe the evolution of CS rates by Robson groups in Uruguay in the last eleven years (2008–2018) using the national SIP database.

## Methods

This research was based data from the SIP National Database. SIP is the result of technical consensus among hundreds of professionals in the Region regularly convened by the Latin American Center for Perinatology, Women’s, and Reproductive Health (CLAP/WR) for review. It is one of the tools Pan-American Health Organization (PAHO) offers to improve the quality of care of mothers and newborns. Among other objectives, it includes clinical care and epidemiological monitoring of data.

We included the records of all women giving birth in Uruguay (pregnancies ≥22 weeks and weighs ≥500 g) with available valid data in the form of termination of childbirth recorded in the SIP database. The period studied starts in the first year where coverage reached ≥80% of the Live Birth Certificate (LBC) issued in the country (Table 1).

**Table 1:** Characterization of births and maternal backgrounds by year

Year	Births (LBC)	Births (SIP)	SIP coverage	CS in LBC	CS in SIP	Rest of the country	Capital city (Montevideo)	Public Health Care sector	Private Health Care sector	Age	Previous pregnancies	BMI	Previous CS
	N	N	%	n (%)	n (%)	n (%)	n (%)	n (%)	n (%)	Mean (sd)	Mean (sd)	Mean (sd)	n (%)
*Total*	*511,846*	*485,263*	*94.8*	*207,182 (40.48)*	*189,872 (43.14)*	*219,881 (45.4)*	*264,886 (54.6)*	*205,649 (42.4)*	*279,118 (57.6)*	*26.7 (6.8)*	*1.2 (1.5)*	*24.2 (5)*	*84,696 (20.2)*
2008	47,104	41,849	88.8	15,729 (33.4)	12,443 (29.7)	17,930 (42.8)	23,919 (57.2)	18,237 (43.6)	23,612 (56.4)	26.6 (6.7)	0.8 (1.4)	23.3 (4.6)	5883 (19)
2009	46,820	42,382	90.5	16,675 (35.6)	13,938 (32.9)	20,211 (47.7)	22,171 (52.3)	20,250 (47.8)	22,132 (52.2)	26.5 (6.8)	1.3 (1.7)	23.4 (4.6)	6410 (19.5)
2010	46,943	42,902	91.4	17,866 (38.1)	15,257 (35.6)	19,355 (45.1)	23,547 (54.9)	19,223 (44.8)	23,679 (55.2)	26.5 (6.8)	1.3 (1.6)	23.6 (4.7)	6712 (19.4)
2011	46,712	42,555	91.1	18,729 (40.1)	16,226 (38.1)	18,889 (44.4)	23,666 (55.6)	18,482 (43.4)	24,073 (56.6)	26.6 (6.8)	1.3 (1.6)	23.8 (4.8)	6948 (19.5)
2012	48,059	45,804	95.3	19,314 (40.2)	18,252 (39.8)	20,590 (45.0)	25,214 (55.0)	19,789 (43.2)	26,015 (56.8)	26.5 (6.8)	1.3 (1.6)	23.9 (4.8)	7709 (19.6)
2013	48,681	47,174	96.9	20,011 (41.1)	19,597 (41.5)	21,333 (45.2)	25,841 (54.8)	19,542 (41.4)	27,632 (58.6)	26.4 (6.9)	1.3 (1.6)	24.2 (5)	8062 (19.9)
2014	48,368	47,449	98.1	21,391 (44.2)	20,087 (42.3)	21,227 (44.7)	26,219 (55.3)	19,030 (40.1)	28,416 (59.9)	26.6 (6.8)	1.2 (1.5)	24.4 (5)	8530 (20.2)
2015	48,926	47,513	97.1	21,072 (43.1)	19,808 (41.7)	21,278 (45.3)	25,742 (54.7)	18,601 (39.6)	28,419 (60.4)	26.7 (6.8)	1.2 (1.5)	24.6 (5.2)	8868 (20.5)
2016	47,058	46,089	97.9	19,843 (42.2)	19,000 (41.2)	21,295 (46.2)	24,794 (53.8)	19,103 (41.4)	26,986 (58.6)	26.9 (6.7)	1.3 (1.5)	24.8 (5.3)	8993 (21)
2017	43,036	42,362	98.4	18,934 (43.9)	18,171 (42.9)	19,809 (46.8)	22,553 (53.2)	17,245 (40.7)	25,117 (59.3)	27.2 (6.7)	1.3 (1.5)	25 (5.4)	8543 (21.4)
2018	40,139	39,184	97.6	17,618 (43.9)	17,089 (43.6)	17,964 (45.8)	21,220 (54.2)	16,147 (41.2)	23,037 (58.8)	27.4 (6.7)	1.3 (1.4)	25.2 (5.5)	8038 (21.4)

Women were characterized based on the data available from births registered in SIP during the period of analysis.

CS rates were calculated by Robson groups for each of the years included, disaggregated by care sector (public/private)^1^ and by geographical area (Capital City/Non-Capital). Women that lacked data on any variables needed to be categorized according to Robson’s classification (parity, previous CS, multiple or singleton pregnancy, weeks of gestation, presentation, and onset of labour) and were classified under the “unclassifiable” category rather than be excluded. Statistical significance was assessed using a trend curve, and an α = 0.05 value was utilized. The software used for data processing and statistical analysis was open source R (version 3.6.1) [24].

All methods were performed in accordance with the Declaration of Helsinki ethical principles. The data used in our study was anonymized.

## Results

The births included were those registered in SIP between 2008 and 2018, as 2008 was the first year in which Uruguay entered more than 80% of the LBCs in the SIP. This resulted in 485,263 records. We removed 15,770 records (3.2%) after pruning records due to missing values in following variables: termination, maternal age, care sector, gestational age or birth weight. The overall CS rate was also calculated on the basis of the LBCs (40.5%) and SIP records (43.1%). The largest difference was 3.7% in favor of a higher rate of CS according to LBCs, observed in 2008 (Table 1).

The country capital (Montevideo) recorded 9.2% more births than the rest of the country (average for the period of the study). The greatest difference (14.2%) was seen in 2008 and the lowest (4.4%) in 2009.Non-profit private health care institutions and private insurances, recorded an average coverage of 15.2% more births in SIP compared to the public sector for the entire period. The difference was lowest (4.4%) in 2009 and highest (20.2%) in 2015. Women’s mean age, parity, and Body Mass Index (BMI) increased slightly between 2008 and 2018, as did the proportion of women with prior CS (*p* < 0.001). Table 1 presents the demographics by year, geographical area (Montevideo and Non-Capital), care sector, and maternal history.

**Fig. 1:**
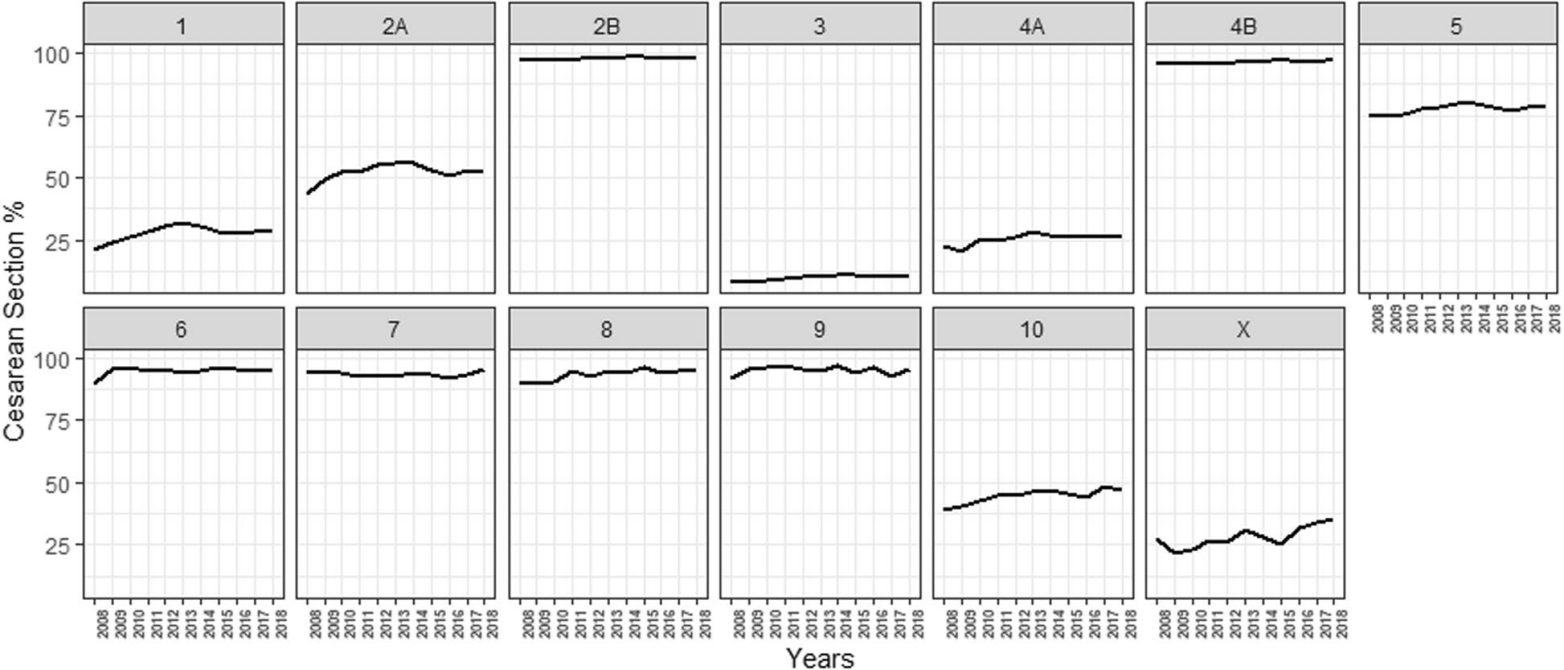
Trends in caesarean section by Robson groups by year

Figure 1 shows the yearly proportion of CS at the country level by Robson groups (Additional Table 1).

Among the groups at lower risk of CS (1 to 4), the highest rates of CS over the period were seen in women in group 2B, followed by those in group 4B. Women in groups 2B, 3 and 4 (A and B) had a significant increase in those 11 years (Significant growth was also observed in groups 5, 8, and 10 (Fig. 1, Additional Table 1).

**Fig. 2:**
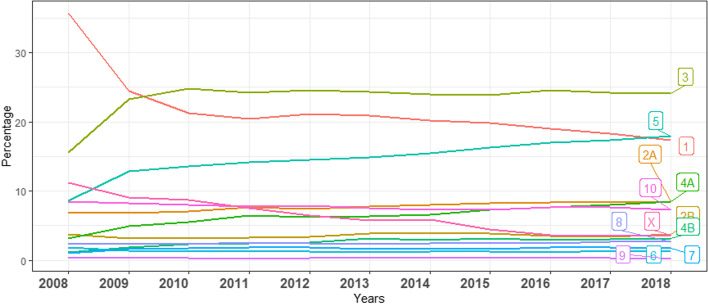
Size of the obstetric population by Robson Group. Relative contribution from 2008 to 2018

The frequency of births by Robson groups over the 11 years, showed a tendency toward an increased relative share of groups 2A, 4A, and 5 (Fig. 2).

**Fig. 3:**
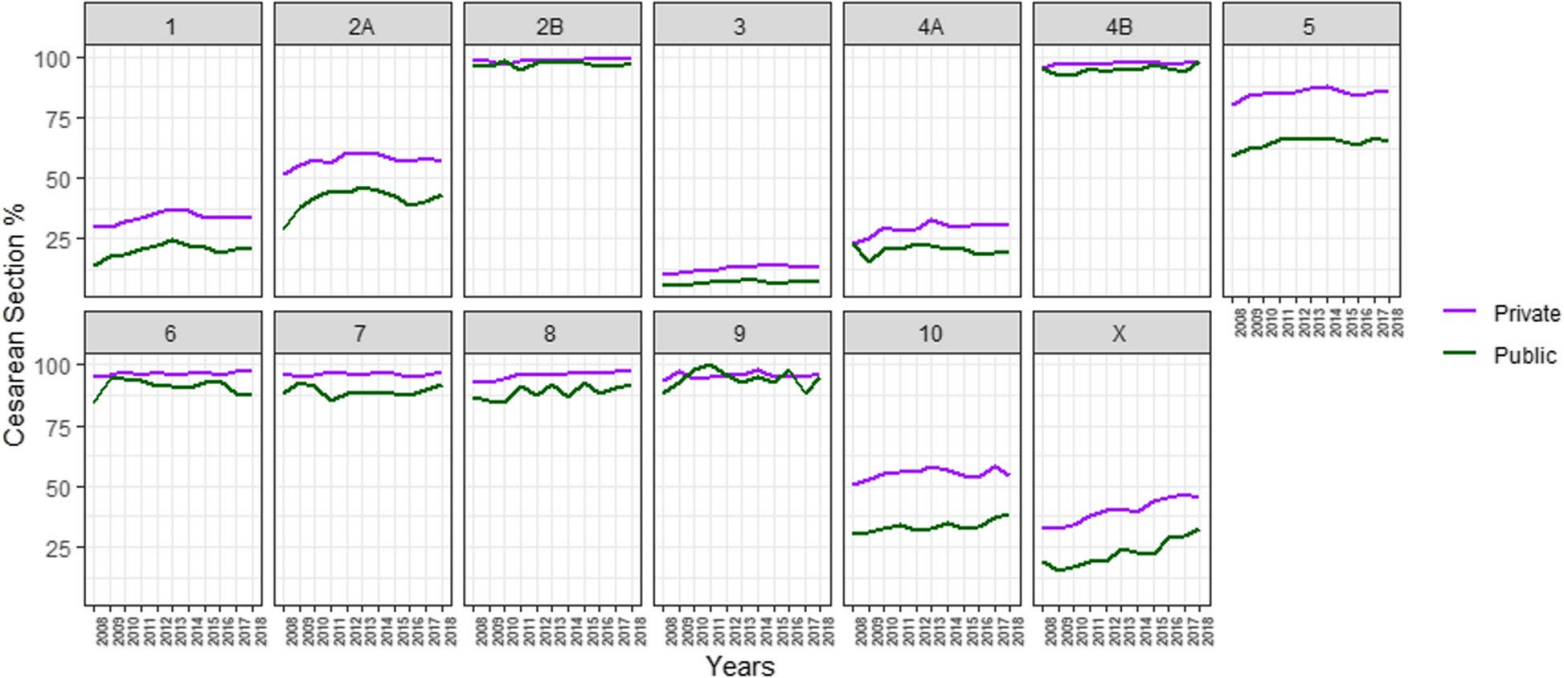
Trends in caesarean sections by Robson groups, years and health care sector

Figure 3 shows the proportion of national CS by Robson’s groups by year and care sector (Additional Table 2). The analysis by care sector showed that the private sector had higher rates of CS for all groups throughout the period, except for women in group 9 where the public sector showed higher rates during three years. Women in group 5 had the largest gap between both sectors for the entire period, with a difference close to 21% for 2018 (85.4% in the private sector and 64.8% in the public sector). The second largest gap was seen in women in group 10, reaching 15.6% (54.2% in private centers versus 38.6% in public centers) in 2018 (Additional Table 2). Women in group 3 were the only ones with CS rates below 15% for the entire period in the private sector, and below 8% for the public sector (Fig. 3). The variations in CS rates in both sectors, showed significant increases over the period in groups 3, 4B and 8. There was also a significant increase in the public sector for group 10 and in the private sector for group 4A (Additional Table 2).

**Fig. 4:**
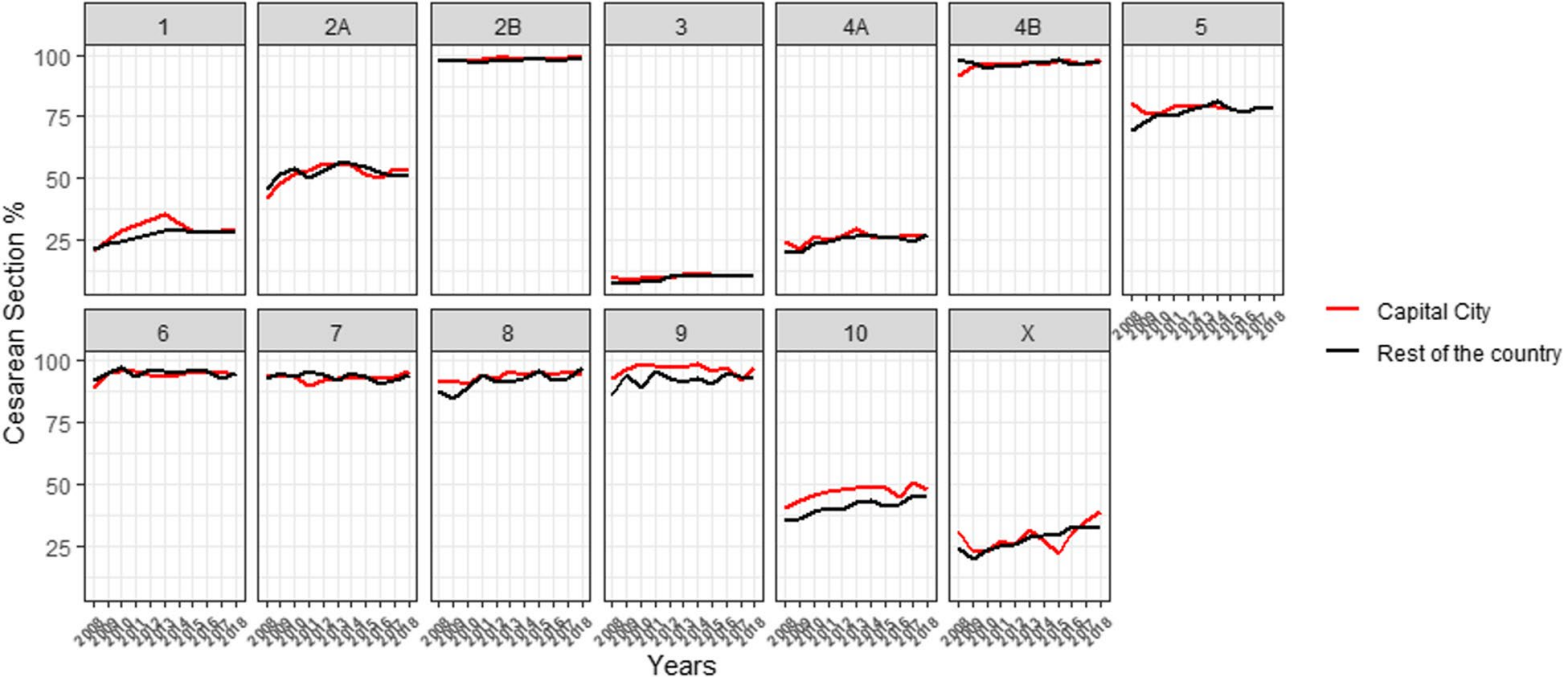
Trends in caesarean sections by Robson group, years and location

In the by-birthplace analysis, there were no differences between the lower risk groups. However, the Non-Capital had lower CS rates in groups 9 and 10 compared to the Capital (Fig. 4; Additional Table 2). Considering variations over the 11 years by place of birth, the Capital City showed a significant increase in the lowest risk groups 2B and 4B, and the Non-Capital in groups 1, 3 and 4A (Additional Table 2). In addition, group 5 showed a significant increase only in the Non-Capital, and groups 8 and 10 showed a significant increase across the country.

When stratifying by both sector of care and birthplace over the research period, the private sector in Montevideo presented the highest rates of CS in the groups with the lowest risk: 1, 2A, 3 and 4A; followed by the private sector in the Non-Capital. Meanwhile, the public sector of Montevideo presented the lowest rates for the 4 groups with the lowest risk of CS (Additional Fig. 1).

Among the Unclassifiable group of women, there was a significant increase in the frequency of CS in the Non-Capital.

The analysis of the relative contribution to the overall CS rate showed that group 5 was the one with the highest contribution, growing steadily over the study period, while there was a decrease in the relative contribution of group 1 to the overall CS rate (Table 2).

**Table 2:** Relative contribution to the overall Cesarean section rate by Robson group, total and by year (%)

Group	Total	2008	2009	2010	2011	2012	2013	2014	2015	2016	2017	2018	Variation	*p* value
1	12.6	23.6	16.9	15	14.7	15.8	15.7	14.5	13.3	12.6	11.9	11.2	−12.4	0.00008427*
2A	8.9	9.2	9.6	10	10.2	10	10.2	10.3	10.1	10.2	10.1	10.2	1	0.7411
2B	7.5	11.7	8.6	8.4	8.1	8.1	9	9	8.9	8.2	7.7	8.2	−3.5	0.2021
3	6.3	4.1	5.4	5.8	5.9	6.1	6.1	6.2	6	6.1	5.7	6.1	2	0.4033
4A	4.4	2.2	2.9	3.8	4.1	4	4.3	4.1	4.4	4.8	4.8	5.2	3	0.1766
4B	6.7	3.2	5.1	5.9	6.2	6.1	7.1	6.6	7.2	6.5	6.7	6.8	3.6	0.1527
5	30.6	19.9	27.1	27.7	27.7	27.8	27.6	28.7	29.6	30.9	31.2	31.8	11.9	0.01061*
6	2.8	5.3	3.9	3.4	3.2	3.2	2.7	2.9	2.8	2.8	3	2.8	−2.5	0.1297
7	4.1	3.1	4.3	4.3	4.1	4.1	3.5	3.5	3.5	3.8	3.8	3.7	0.6	0.7506
8	6.1	6.4	5.9	5.6	6.3	5.6	5.1	5.4	5.8	5.5	5.9	5.8	−0.6	0.8738
9	0.7	0.8	0.8	0.9	0.6	0.6	0.6	0.8	0.6	0.7	0.6	0.5	−0.3	0.6706
10	9.1	10.5	9.5	9.2	8.9	8.6	8.1	8	7.8	7.9	8.6	7.7	−2.8	0.2936

* indicate significant values

Neonatal outcomes were analyzed among the groups at lower risk of CS which had a significant increase on their CS rates over the period. No significant differences were observed in 5-minute Apgar scores < 7 over this period. However, there was a decrease in neonatal death rates over time at hospital discharge in groups 1 (from 0.000727 to 0.000619, *p* = 0.043) and 3 (from 0.001119 to 0.00045, *p* = 0.034), as well as in stillbirths in group 4A (from 0.004201 to 0.002879, *p* = 0.031) (Additional Figs. 2, 3 and 4).

**Fig. 5:**
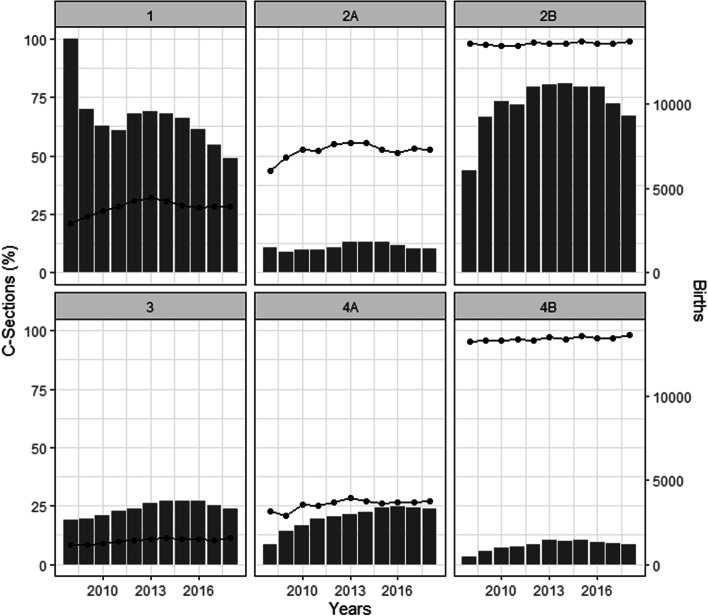
Percentage of caesarean sections and number of births by Robson groups between 2008 and 2018

There was a trend toward growth in the groups at lower risk of CS (2B to 4). Those groups also showed significant increase in the CS rates (2B, 3, 4A and 4B) (Fig. 5, Additional Table 1).

## Discussion

Globally, CS rates are increasing, even in groups of women who would be expected to have a lower risk of requiring CS [5, 25]. The percentage of births that occur through CS in LAC reaches 40.5% [2]. Our study shows that Uruguay is no exception to this trend, and that over the years the medicalization of childbirth has increased similarly to other LAC countries [4].

The analysis of CS rates by Robson groups enabled us to rule out parity and the presence of previous CS as potential explanations for CS rate increase, because the CS rates increased regardless of an increase in these risks factors for CS throughout the period (Table 1).

Groups 6 through 9, which include nulliparous and multiparous patients with singletons in breech, or transverse presentation, or twins with CS rates typically over 90% actually contributed less than 6% each in 2018. The high rates in groups 6, 7 and 9 reflect the adoption of the recommendation that emerged from the systematic review on breech labour [26, 27]. Yet, the evidence available does not justify the high rates of CS observed in women with multiple pregnancies, including women with previous uterine scars (group 8) [28]. In addition, there is no justification for the significant contribution to the overall rate of groups 1, 2A, 2B, 3 and 4B for 2018, considering their low risk. Group 5 had the highest relative contribution in 2018 and was the group with the largest increase in terms of relative contribution during the included years (Table 2). This shows that the increase generated over time in the groups of nulliparas at lower risk has led to an increase in the number of patients with caesarean scar, a simple consequence of the increase in the overall rate of CS, largely concentrated among women in group 5.

The results of this study are comparable with those obtained in the developed world. A study in Canada found that the group with the highest contribution to the overall CS rate was also group 5 [29]. Consistently, a study in Brazil reported group 5 leading the CS relative contribution rates; and a review stated that previous CS is the primary indication in approximately 30% to current CS [30, 31]. Our study shows that while, in 2018 this group accounted for approximately 20% of the obstetric population (Fig. 2), one-third of all the women undergoing a CS were in this group. The implementation of non-clinical interventions targeted at organizations, facilities and systems can affect CS rates [32, 33].

Earlier studies have reported excessive interventions in high-income countries, particularly in the private sector. The increase in facility use has been accompanied by widespread over-medicalization of birth, particularly in high and middle-income countries, calling the phenomenon “too much, too soon” [34], These countries passing through the obstetric transition often implement unnecessary or inappropriate obstetric interventions in health facilities, which is a cause for concern [25], and reflect weak enforcement capacity and low compliance to evidence-based practices. The overuse of unnecessary CS in low-risk women cannot be associated with the improvements observed in neonatal outcomes since perinatal interventions with an impact on neonatal health have been incorporated over time. The analysis of the proportions of CS in the groups with the lowest risk of receiving a CS by sector of care, reveals differences to the detriment of women in the private sector. In the last year, these differences ranged between 14.6 and 11.1% (for groups 1 and 2 respectively). Although this study did not incorporate information prior to 2008, we see that from that year on there was a slight trend towards an increase in the number of births taking place in the private sector, a phenomenon that can be explained by the changes in the health care system. Considering that in the private sector the criteria for the indication of CS to patients with the same obstetric risk is “laxer”, the migration of users from the public to the private sector would have increased the number of potential CS recipients.

There are multiple factors that affect and explain the increasing rates of CS where the frequency is greater than needed. The decision to use CS is driven by three broader, interconnected categories: 1) childbearing women, families, communities, and the broader society; 2) health professionals; and 3) health-care systems, financing, and organizational design and cultures. These categories include economic, logistic, the culture of the women and their families, professionals views, organization of the health care system, and funding structures or incentives [9, 35, 36].

Uruguay’s health care system is organized in such a way that only a minority wealthy, privileged sector of clients is able to choose the doctor that will take care of their delivery, even women assisted in private services have to pay if they want to choose a particular doctor for childbirth. The substantial majority of births are left in the hands of the obstetricians on duty. Thus, financial incentives are less likely for on-call obstetricians to expedite births. Some studies have reported the lack of skills to conduct a vaginal birth [37–39], perception of CS as beneficial [37, 40], the belief that women prefer a CS or the perception that women are not capable of having a vaginal birth [41–43] as reason for the high rates of CS. However, according to a recent review, only a minority of women from different countries and situations stated a preference for CS as a mode of delivery [44]. Many studies have even reported that women claimed they lacked autonomy over birth-related decisions, including the experiences of several women who said they had initially rejected the option of a CS, only to be eventually convinced to undergo CS by the doctor in charge at the time [45–50]. In Uruguay, it would be important to review and strengthen the implementation of existing clinical guidelines on the management of induction of labour and scheduling of caesarean delivery. In addition, the provision of comprehensive health education and counseling during antenatal care should be a priority, as recommended by WHO.

### Strengths and limitations

Our analysis has some limitations. Due to SIP coding constraints, we were unable to discern clients covered by private health insurance (who account for about 3% of the total number) or clients of the university hospital. Both cases are likely to present sharp differences with the rest of the population.

Considering SIP is a clinical record with assistance purposes, there is a lack of information on “non-clinical variables” such as those that drive the increase of CS rates in the absence of clinical indication.

This is the first trend analysis in Uruguay at the national level using the Robson classification with high coverage of birth due to the well-established SIP as a standard for data collection during pregnancy and birth. We obtained a high-quality database with low missing rates for the calculation of the Robson categories. Thanks to the nationwide implementation of the system, combined with the universalization of institutional childbirth it is possible to obtain national indicators by subsectors and geographies and also comparable over time; this is highly beneficial for clinical practice, research, audits, management, and evaluation of health care services. This software allows alerts about situations that differ from what would be expected, such as to anticipate the risk of CS and obtain indicators by Robson groups in real time. The monitoring of CS rates by Robson groups is a strategy that allows health decisions to be made, while ensuring the comparability of information. It is important to allocate human and budgetary resources to maintain and improve the systematization and entry of registries into the system, allowing the continuity of epidemiological surveillance of perinatal and maternal health in the countries of the region.

## Conclusions

The results obtained in this study support the view that the implementation of interventions aimed at reducing CS in the groups with lower obstetric risk in Uruguay is warranted. These groups are currently responsible for the steady increase of patients presenting with a scar in the uterus, and the ensuing unjustifiable increase in the rates of CS over the years. Strategies for successful implementation of clinical and non-clinical interventions to reduce CS, where overuse is common, are urgently needed. We suggest the design of multifaceted, context-specific interventions oriented to all “stakeholders” implementing formative research that addresses the concerns, limitations and strengths of each situation following a broad discussion with professionals and with the active participation of women. Things need to change to reduce unnecessary risks and expenses, and essentially to reposition women and their families as the key players at childbirth.

## Supplementary Information

**Additional file 1.**

**Additional file 2.**

**Additional file 3.**

**Additional file 4.**

**Additional file 5.**

**Additional file 6.**

## Data Availability

Data cannot be shared publicly because of confidential issues. Data are available from the Department of Vital Statistics, Ministry of Health of Uruguay. Institutional Data Access (contact Adriana Misa_ amisa@msp.gub.uy), for researchers who meet the criteria for access to confidential data.

## Abbreviations

CS: Cesarean section
SIP: Perinatal Information System
WHO: World Health Organization
LAC: Latin America and the Caribbean
IQR: Interquartile Range
CLAP/WR: Latin American Center for Perinatology, Women’s and Reproductive Health
PAHO: Pan-American Health Organization
LBC: Live Birth Certificate
BMI: Body Mass Index

## Footnotes

1 By private institutions we are referring to IAMCs – non-profit private institutions-.
