# Supplementary figures and images for: Cesarean section in Uruguay from 2008 to 2018: country analysis based on the Robson classification. An observational study
Source: BMC Pregnancy Childbirth. 2022 Jun 7;22:471. doi: 10.1186/s12884-022-04792-y (PMC9175367; doi:10.1186/s12884-022-04792-y)

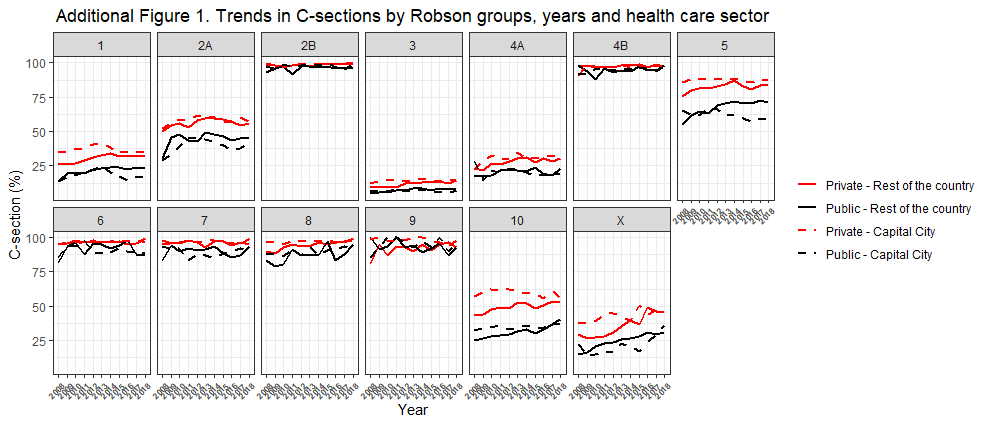

Supplement: Supplementary file 3 — Additional file 3. [file 12884_2022_4792_MOESM3_ESM.png]

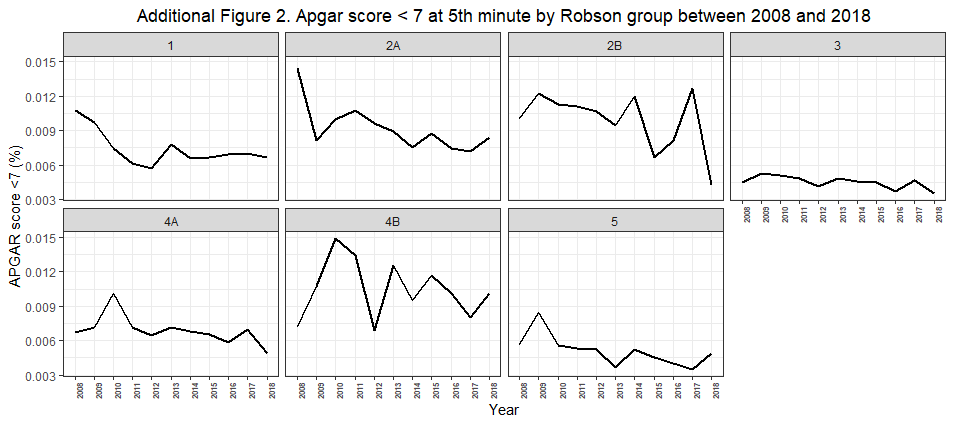

Supplement: Supplementary file 4 — Additional file 4. [file 12884_2022_4792_MOESM4_ESM.png]

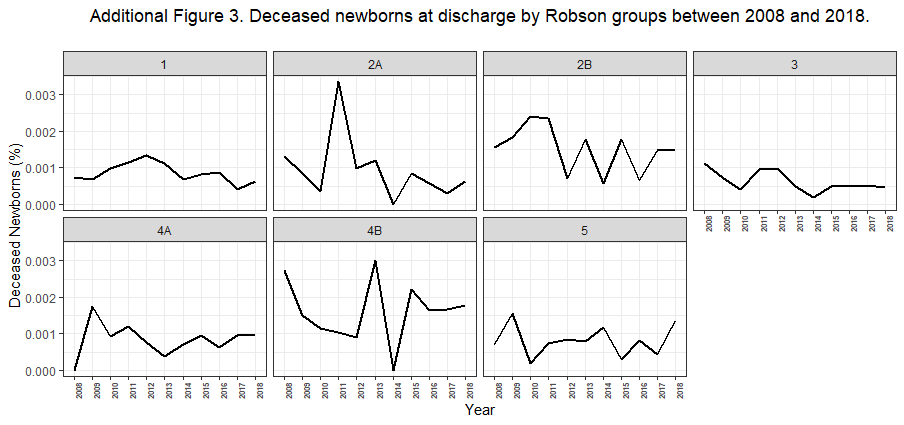

Supplement: Supplementary file 5 — Additional file 5. [file 12884_2022_4792_MOESM5_ESM.png]

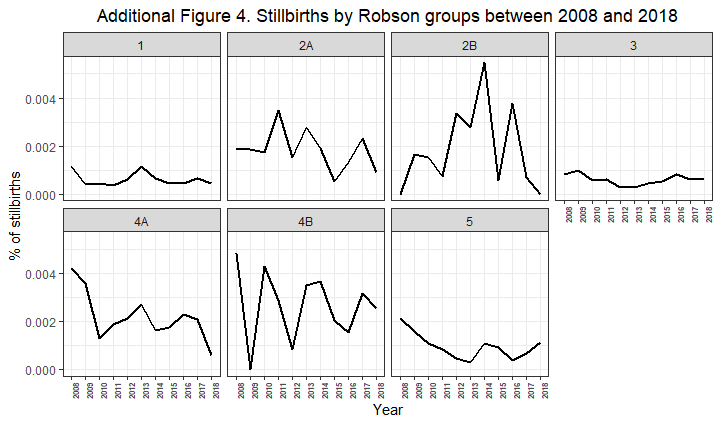

Supplement: Supplementary file 6 — Additional file 6. [file 12884_2022_4792_MOESM6_ESM.png]
